# Study on Wear and Corrosion Resistance of Ni60/WC Coating by Laser Cladding on Reciprocating Pump Plunger: Comparison with Flame-Sprayed Plungers

**DOI:** 10.3390/ma17215183

**Published:** 2024-10-24

**Authors:** Xiaogang Wang, Jingjing Qi, Hao Zhang, Ning Zhao, Zhangbin Shao, Shuyao Wang

**Affiliations:** 1 Technology Inspection Center of Shengli Oilfield, SINOPEC, Dongying 257061, China; t-wangxg995.slyt@sinopec.com (X.W.); qijingjing533.slyt@sinopec.com (J.Q.); zhanghao.slyt@sinopec.com (H.Z.); zhaoning.slyt@sinopec.com (N.Z.); shaozhangbin.slyt@sinopec.com (Z.S.); 2 College of Mechanical and Electrical Engineering, China University of Petroleum (East China), Qingdao 266580, China

**Keywords:** reciprocating pumps, plunger surface modification, laser cladding, Ni60/WC coating, wear and corrosion resistance

## Abstract

Reciprocating pumps are widely used in the current oil extraction process, and the plunger is a vulnerable part of these pumps that directly determines the service life of the reciprocating pump. To improve the service life of plungers, Ni60/WC coatings were applied to the surface of 45-steel plungers via laser cladding technology to improve wear and corrosion resistance. Defect-free and dense Ni60/WC coatings were successfully applied to the plunger surface with strong metallurgical bonding between the coating and the substrate. The coating consists mainly of a γ-(Ni, Fe) phase, which contains isotropic and isotropic-like crystals, dendritic crystals, and columnar crystals in the top, middle, and bottom regions of the coating, respectively. The service performance of the laser cladding coating was compared to the flame-sprayed plunger, which is widely used, and the laser cladding coating has a microhardness of up to 821.8 HV_0.5_, which is higher than that of the flame-sprayed coating (545.5 HV_0.5_) and the 45-steel substrate (200 HV_0.5_). The laser cladding coating has a lower friction coefficient and a smaller volumetric wear rate, and the corrosion current density and corrosion rate in the NaCl solution are 2.52 × 10^−7^ A/cm^2^ and 2.96 × 10^−3^ mmPY, respectively, which indicates superior corrosion resistance to the flame-sprayed coating and the substrate. The laser cladding of reciprocating pump plunger surfaces has a significantly improved comprehensive performance and is a promising way to increase the service life of reciprocating pumps.

## 1. Introduction

**Figure 1:**
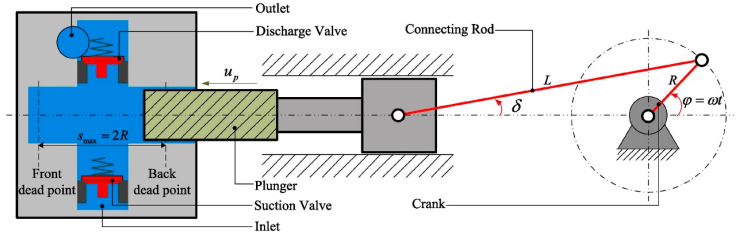
Schematic diagram of the reciprocating pump [1].

Reciprocating pumps convert the rotary motion of the motor into the reciprocating linear motion of a volumetric pump as the reciprocating motion of the plunger constantly changes the volume inside the chamber, enabling the suction and discharge of liquid [1]. Reciprocating pumps can generate high working pressure and are widely used in oil extraction processes. However, the plunger, as a key part of the reciprocating pump, directly determines its service life and reliability, which, in turn, affects the production safety and economic benefits of the oilfield. Reciprocating pumps (including wellhead booster pumps) currently in service in the Shengli Oilfield total more than 1200 units, and in the two-year period from 2020 to 2022, 2057 reciprocating pump plungers were replaced, with the accumulated cost of plunger parts exceeding CNY 10 million. As shown in Figure 1, the plunger forms a friction vice with the packing during the normal operation of the reciprocating pump to ensure lower leakage at higher pressures or even zero leakage. Therefore, the sealing performance and service life of the plunger affect the operating performance of the reciprocating pump [2]. The working principle of reciprocating pumps means that the main failure modes of the plunger are surface wear failure, corrosion failure, and corrosion–wear coupled failure. Therefore, improving the wear resistance and corrosion resistance of the plunger surface is essential to improve the service life of reciprocating pumps.

Laser cladding technology involves the use of a concentrated laser heat source to irradiate the surface of a metal material, forming a molten pool; the metal powder is then coaxially delivered to the molten pool on the surface of the material, and after solidification, a metallurgical bond is formed with the metal material coating. The preparation of high-performance coatings on the surface of vulnerable parts by laser cladding technology to enhance the damage resistance of components is a key method to improve the overall performance of mechanical components [3]. Laser cladding technology offers the advantages of low heat input, low substrate dilution, and high processing efficiency, with great potential for industrial applications [4] and widely used in aerospace, petrochemical, transportation, and other fields [5,6]. Ding et al. [7] prepared Inconel 625 coatings on the surface of 27SiMn steel by optimizing the conventional laser cladding technique, resulting in a grain-refined microstructure and achieving further improvements in coating microhardness, wear resistance, and corrosion resistance. To improve the corrosion resistance of Q235 steel, an Fe5Cr5SiTiCoNbMoW high-entropy alloy coating was prepared on the surface using laser cladding technology. The results show that the microhardness of the coating is five-times that of the substrate, and its corrosion resistance in NaCl solution is superior to that of 304 stainless steels [8].

Among the material systems for coating preparation by laser cladding, Ni60 alloy powder offers high hardness, good machinability, and excellent corrosion resistance, and it also exhibits high strength and oxidation resistance under high-temperature conditions, making it a widely used material. Lu et al. [9] successfully prepared Ni60 coatings on Q235 substrates by laser cladding using optimized process parameters; the microhardness of the coatings reached 499.4 HV, and the wear resistance of the coatings was significantly improved compared to the substrate. Studies have shown that WC is widely used in laser cladding technology due to its high hardness, good wear resistance, and good wettability, and the addition of reinforcing particles, such as WC, to metal-based coatings can improve the mechanical properties of the coatings [10,11,12]. Therefore, adding WC particles to Ni60 alloy powder as the raw material for laser fusion coating prepared on the surface of critical parts can combine the excellent properties of the two so that the usability of reciprocating pump plungers can be significantly improved [3,13]. Chen et al. [14] prepared Ni60 composite coatings with nano and micron WC particles on the surface of an impeller via laser cladding technology. The study of the coatings’ microhardness friction and wear properties showed that the WC particles had a significant reinforcing effect on the coatings, and the microhardness and wear resistance were improved. Feng et al. [15] prepared Ni60/WC coatings on the surface of the Ti6Al4V substrate, and it was found that there was good metallurgical bonding between the coating and the substrate. The average microhardness of the coating was 2.75–3.13-times that of the substrate, and the mass wear rate was reduced by 60.1–79.7%. Miao et al. [16] prepared Ni60/WC/La_2_O composite coatings on Cr12MoV substrate via the laser cladding technique. The results showed that WC particles were partially dissolved to form new carbides with lower hardness, while adding La2O particles led to a coating microhardness as high as 1024.3 HV. The abrasion resistance of the coatings was reduced by 88.8% compared with that of the substrate. Qunshuang et al. [17] prepared WC particle-reinforced nickel-based composite coatings on a Q550 steel substrate, where WC particles were partially dissolved and reacted with molten Ni60 powder due to the irradiation of a laser heat source to form a special eutectic structure composite coating. A dry sliding wear tester characterized the wear resistance of the coatings, and it was found that the coatings exhibited excellent wear resistance, which was about 6.8-times that of the base steel.

There are few reports on the application of laser cladding technology on the vulnerable parts of reciprocating pumps, and there are fewer related application cases. In this study, Ni60 powder and a small amount of WC particles are selected as raw materials for laser cladding of the Ni60/WC coating on the surface of a 45-steel plunger of a reciprocating pump, and the microstructure, microhardness, abrasion resistance, and corrosion resistance of the coating are studied. The microstructure, microhardness, sliding wear resistance, and corrosion resistance of the coating are analyzed and compared with those of the flame-sprayed Ni60 plungers, which are currently widely used. We provide a specific reference value for the application of laser cladding technology in oilfield equipment, especially in the preparation of the Ni60/WC coating on the surface of the plunger, which is a fragile part of the reciprocating pump, to improve the service life of the plunger.

## 2. Experimental Procedures

Ni60 spherical alloy powder with a particle size range of 53~105 μm (D50 = 101.2 μm, Chengdu, China) and WC (D50 = 96 μm, Chengdu, China) spherical particles with a purity of 99.9% were selected as the raw materials for the laser cladding of Ni60/WC coatings. A 45-steel plunger, one of the reciprocating pump plunger types, with a diameter of 38 mm was selected as the base material, and the outer diameter of the plunger was machined to 36.8 mm using a 2000-mesh grinding wheel (Machining from drawings, Qingdao, China) and then used directly for surface laser cladding. The chemical compositions of the substrate and Ni60 alloy powder used to prepare the Ni60/WC coatings are given in Table 1. The weight percentage of Ni60 alloy powder and WC particles was 9:1, and the desired dispersion was obtained by mixing the two powder particles under gravity [18] for a mixing time of 3 h. The two powders’ particles were used to prepare the Ni60/WC coatings.

**Figure 2:**
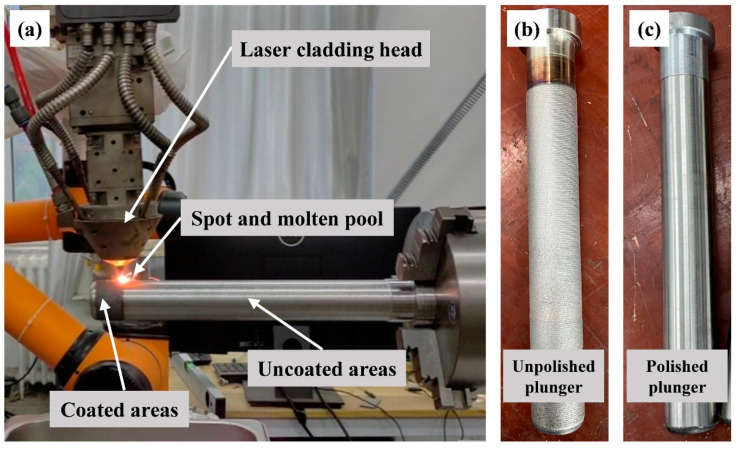
(**a**) Experimental platform and equipment for laser cladding of Ni60/WC coating. (**b**) Morphology of the untreated plunger after laser cladding. (**c**) Morphology of the plunger after grinding and polishing the Ni60/WC coating via laser cladding.

The experimental platform for preparing Ni60/WC coating on the surface of a plunger is shown in Figure 2, which mainly consists of a fiber laser (MAX, Shenzhen, China), a coupled coaxial nozzle head (Precitec, Shanghai, China), a robotic arm (STEP, Shanghai, China), and a rotary chuck (Shenzhen, China). The fiber laser generates a high-energy laser, and the coaxial nozzle head is used to deliver the Ni60/WC powder output from the powder feeder to the molten pool generated on the substrate surface. The mechanical arm and rotary chuck are used to control the linear motion of the laser heat source and the rotary motion of the plunger, respectively. The detailed process parameters for the preparation of Ni60/WC coatings by laser melting are shown in Table 2. Argon was used as the protective gas in the laser cladding process to isolate the liquid metal in the molten pool generated by laser irradiation from the ambient oxygen. The finished morphology of the laser melting coating on the plunger surface is shown in Figure 2b. Although the expected lap ratio was used to prepare the coating, it could not be used directly in the reciprocating pump. As shown in Figure 2c, the laser-melted plunger surface was machined by grinding and polishing to obtain a plunger that could be used directly in a reciprocating pump, and the outer diameter of the polished plunger was 38 mm.

**Figure 3:**
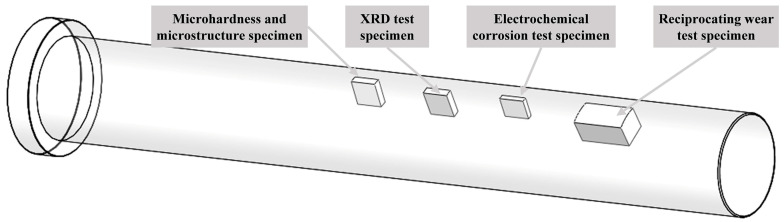
Selection of specimens for characterizing the properties of Ni60/WC coatings after laser cladding.

**Figure 4:**
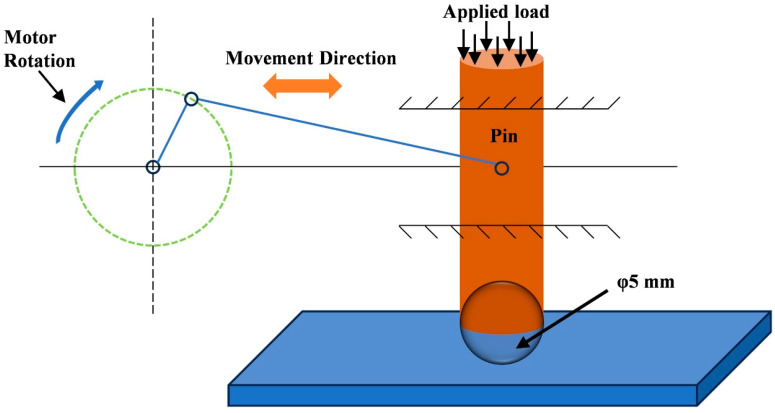
Schematic of reciprocating wear test of the plunger with laser cladding Ni60/WC coating [14].

The experimental samples for microstructural analysis, phase composition analysis, and performance testing were cut from the ground and polished plungers using a wire-cutting machine, as shown in Figure 3. The polished specimens were etched using aqua regia solution (Vol (HCl): Vol (HNO_3_) = 3:1), and the standard metallographic samples prepared were used to analyze the microstructural morphology and chemical composition. The microstructure and chemical composition of the laser-melted Ni60/WC coatings were investigated using scanning electron microscopy (SEM, ZEISS, MERLIN Compact, Oberkochen, Germany) and energy-dispersive spectroscopy (EDS, OXORD, X-Max^N^, Oxford, UK), respectively. The Ni60/WC coatings were analyzed for phase composition using an X-ray diffractometer (XRD, Rigaku, Ultima IV, Tokyo, Japan) with Cu-targeted Kα diffraction, and the 45-steel substrate and coated specimens were tested separately at a scanning speed of 5°/min in the range of 20° to 90°. A Vickers hardness tester (HV-1000, Laizhou, China) was used to test the microhardness of laser cladding Ni60/WC coatings and flame-sprayed coatings with an applied load of 500 g and a holding time of 10 s. The wear resistance of the coatings was tested using a reciprocating wear tester (MFT-4000, Lanzhou, China) with a wear length of 5 mm and a load of 10 N, respectively. The counterpart of friction with the coating was a GCr15 stainless-steel ball with a diameter of 5 mm, and the friction coefficient was recorded during 30 min of friction and wear. The principle of the reciprocating wear test is shown in Figure 4. A three-electrode electrochemical analysis system (CS350M, Wuhan, China) was used to analyze the corrosion resistance of 45-steel substrates, laser cladding, and flame-sprayed coatings. The corrosion solution was a 3.5 wt.% NaCl solution; saturated calomel electrode, platinum electrode, and test specimen were used as the reference, counter, and working electrode, respectively.

## 3. Results and Discussion

### 3.1. Morphology and Phase Composition

**Figure 5:**
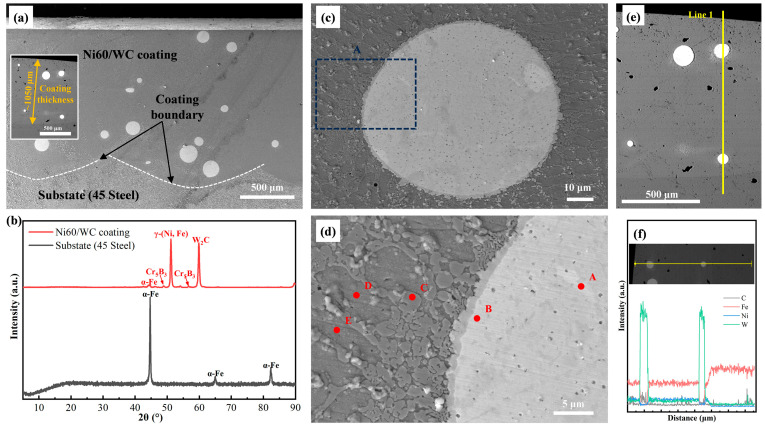
(**a**) Macroscopic morphology of the axial section of the plunger after laser cladding coating; (**b**) XRD spectra of Ni60/WC coating via laser cladding and 45-steel substrates; (**c**) SEM image of WC particles inside the Ni60/WC coating via laser cladding and (**d**) high-magnification image of region A in Figure 5c; (**e**) Cross-sectional morphology perpendicular to the axial direction; (**f**) Results of the elemental distribution of line 1 in Figure 5e.

To analyze the macroscopic morphology of the laser-melted Ni60/WC coating and the metallurgical bonding effect with the 45-steel substrate, the Ni60/WC coating was observed at low magnification. Figure 5a shows the macroscopic morphology of the Ni60/WC coating along the axial cross-section of the plunger. It can be found that spherical WC particles are dispersed inside the coating, and the coating is free of defects such as microcracks and pores. The inset in Figure 5a shows the coating thickness measurement within a cross-section perpendicular to the axial direction. The thickness of the laser-melted cladding layer is 1.05 mm, which is much greater than the allowance on one side (0.6 mm on one side), indicating that the coating and the substrate are fully diffused under the laser heat source, and the metallurgical bonding between the coating and the substrate exhibits excellent results. In addition, the volume fraction of WC particles in the laser cladding coating is lower than expected, the statistical results show that the volume fraction of WC particles in Figure 5a is ~4.2%, and the densities of WC particles and Ni60 powder are 15.63 g/cm^3^ and 8.9 g/cm^3^, respectively. The calculation results show that the mass percentage of WC particles in the laser cladding coating is 7.7%. The experimental results below the design values indicate that some WC particles decompose at high temperatures. This is mainly related to the fact that the energy is more concentrated at the center of the laser heat source than at the edges, and the resulting energy inhomogeneity also leads to the formation of a curved boundary at the bonding of the coating with the substrate.

An SEM image of the WC particles inside the laser cladding Ni60/WC coating is shown in Figure 5c, which reveals that there are no inclusions of pores near the WC particles, and the particle phase has a metallurgical bonding effect with the Ni60 alloy [19]. Figure 5d shows an enlarged view of region A in Figure 5c. The organization near the WC particles exhibits the phenomenon of average growth along the outer edges of the particles, which further indicates that elemental diffusion occurs at the edges of the Ni60 alloy and the particle phase and the diffusion between the elements is beneficial to the metallurgical bonding effect. The elongated grains growing along the radial direction of the WC particles indicate that the WC particles become the nucleation site of the liquid metal, and the grains grow further in the direction of the heat gradient after nucleation. In addition, based on the morphology of the WC particles, it was found that irradiation by the laser heat source did not lead to the destruction and fracture of the particles and that their sizes conformed to the range of particle sizes of the raw material. The chemical compositions of different regions at different locations (A–E in Figure 5d) are shown in Table 3. The EDS results at points A and B demonstrate that the white spherical particles in Figure 5 are un-melted WC particles. The W/C ratio in the chemical composition of point A is closer to 1 than that of point B, which is near the edge of the WC particles. This indicates that elemental diffusion occurred between the WC particles and the melted Ni60 alloy after irradiation by the laser heat source. The results of EDS line scans of the coatings within a cross-section perpendicular to the axial direction, as shown in Figure 5e,f, provide further evidence of the elemental diffusion behavior, with the element Fe increasing with increasing distance from the coating surface and the coating containing more Ni.

To analyze the physical phase composition of the laser-melted Ni60/WC coating, the XRD diffraction patterns of the 45-steel matrix and the Ni60/WC coating are shown in Figure 5b. It can be found that the 45-steel matrix is mainly composed of α-Fe, and the lamellar α-Fe phase is an essential phase for 45-steel to exhibit a pearlitic structure at room temperature. Compared to the matrix, the laser-melted Ni60/WC coating exhibits high-intensity diffraction peaks of γ-(Ni, Fe) phase, indicating that the γ-(Ni, Fe) phase is the parent phase of the coating, which is consistent with the results found by Qunshuang et al. [20] and Venkatesh et al. [21]. The absence of detectable diffraction peaks of WC particles in the XRD diffraction spectra of the coatings suggests that WC was partially dissolved after laser irradiation and that the WC particles were only present in the interior of the coatings and not on the surface.

### 3.2. Microstructure

**Figure 6:**
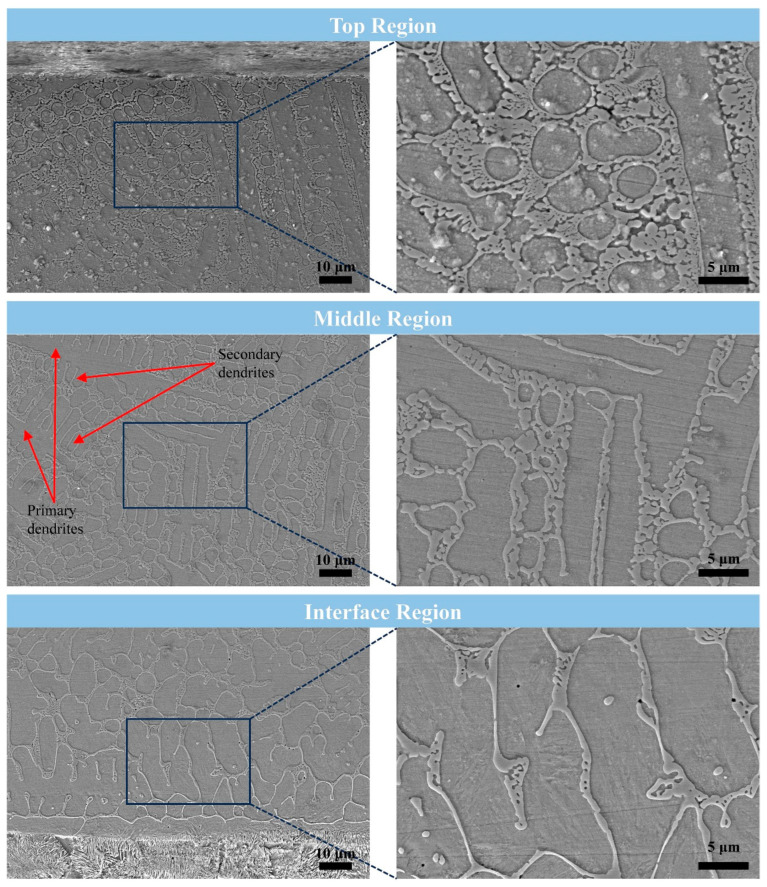
Microstructure of different Ni60/WC coating regions via laser cladding on the surface of 45-steel plunger.

The microstructures of the Ni60/WC coating via laser cladding in different regions are shown in Figure 6. It can be found that the top region of the coating is dominated by equiaxial and equiaxial-like crystals with smaller grain sizes, which is mainly due to the accelerated solidification of the molten metal on the surface of the coating after laser cladding by thermal convection between the surface of the coating and the environment. As a result, the rapid solidification process inhibits the further growth of the grains, leading to a microstructure with smaller grain sizes in the top region of the coating. The fast heating and cooling of the laser heat source are the main reasons for the higher solidification rate of the liquid metal, which is the main feature of the laser melting and cladding technology [6,22]. In the central region of the coating, the grains show a typical dendritic crystal structure, with more considerable lengths for primary dendrites and smaller ones for secondary dendrites. The growth direction of the primary dendrites usually coincides with the direction of the thermal gradient, indicating that the inner region of the coating has more time for grain growth than the top region. This is mainly because the interior of the molten pool formed by laser irradiation usually has the longest solidification time. The liquid metal solidification process is affected by the temperature gradient. The grain growth direction of the nucleation is consistent with the direction of the temperature gradient, which shows the growth along the perpendicular direction of the fusion line between the coating and the substrate. In the bottom region of the coating, the cytosolic crystals show the phenomenon of elongation along the vertical direction of the fusion line between the coating and the substrate, and the grain size of the cytosolic crystals is significantly larger compared with that of the top region. Small grains were found at the grain boundaries of the cytolysis, mainly composed of tiny grains that did not have enough time and space to grow. Analysis of the chemical composition of the grains and grain boundaries shows that the dendrites and cytosols are mainly composed of Ni, Fe, and Cr. In contrast, the grain boundaries are primarily populated with the elements B and W from the melting decomposition of WC particles. Due to the low proportion of WC particles added in Ni60 alloy and the decomposition of only some WC particles, the decomposed W elements are polarized at the grain boundaries, and the C elements do not have an apparent polarization phenomenon in the coating.

### 3.3. Microhardness Distribution

**Figure 7:**
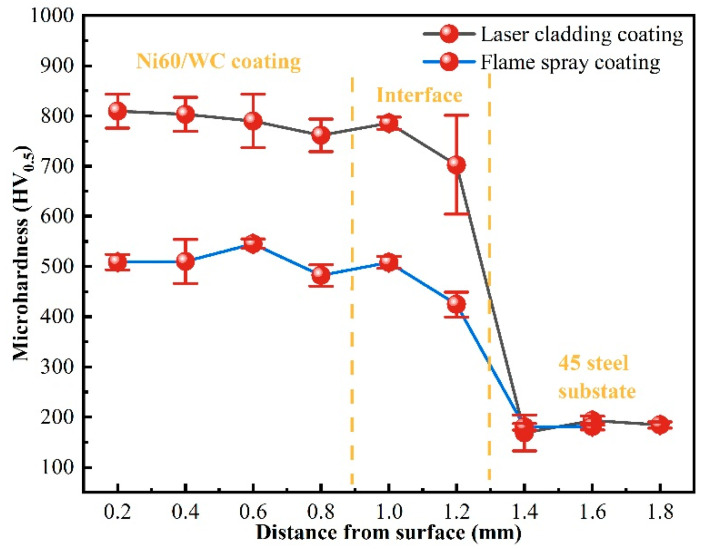
Microhardness distribution of Ni60/WC coating via laser cladding on the surface of the plunger and flame-sprayed coatings.

The results of the microhardness distribution of the laser cladding Ni60/WC coatings within the cross-section are shown in Figure 7, and it can be found that the coatings and the 45-steel substrate have significantly different microhardness values, with the microhardness curves showing a stepped shape. The Ni60/WC coating has the highest microhardness, followed by the interface between the coating and the substrate, and the 45-steel substrate has the lowest microhardness, which is a typical feature of the microhardness distribution of the coatings prepared via laser cladding technology [23,24]. Figure 7 also shows the results of the distribution of the microhardness of flame-sprayed coatings within the cross-section, and the hardness higher than 500 HV_0.5_ is one of the reasons why they are widely used in oilfield equipment. High microhardness is an inherent property of Ni60 alloy materials, and the microhardness can be further increased with the addition of WC particles. On the one hand, the WC particles are ceramic particles, which show great microhardness values, but, on the other hand, the addition of WC particles provides more nucleation sites in the liquid molten metal in the molten pool after irradiation by the laser heat source, which can serve the purpose of grain refinement. The highest microhardness values for the laser cladding and flame-sprayed coatings were 821.8 and 545.5 HV_0.5_, respectively, which were 310.9% and 172.8% higher compared to the 45-steel substrate (~200 HV_0.5_). Therefore, laser cladding coatings show more competitive advantages in microhardness. In addition, the microhardness of the Ni60/WC coating within the cross-section exhibits a larger top region than the middle region, which corresponds to the microstructure shown in Figure 6, where the finer microstructure in the top region of the coating further improves the microhardness of the coating. This result can be attributed to the addition of WC particles, leading to microstructure refinement and a hard-phase diffuse distribution strengthening effect [25].

### 3.4. Wear Resistance

**Figure 8:**
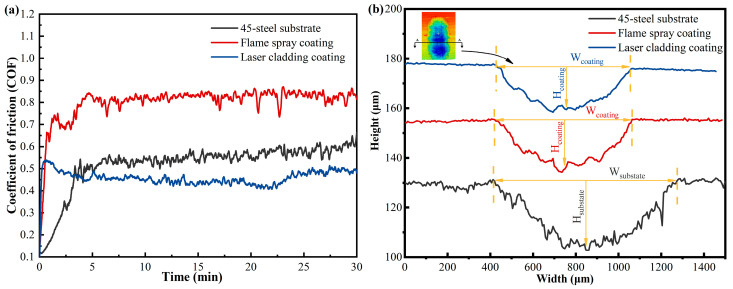
(**a**) Reciprocating coefficient of friction of laser cladding Ni60/WC coating on a plunger, flame-sprayed coatings, and 45-steel substrates; (**b**) cross-sectional profiles of laser cladding Ni60/WC coating, flame-sprayed coatings, and 45-steel substrates after wear.

The friction coefficients as a function of time for the laser cladding Ni60/WC coating, flame-sprayed coating, and 45-steel substrate during reciprocating frictional wear are shown in Figure 8a, and it can be found that the friction coefficient of the 45-steel substrate increases gradually with the reciprocal wear process. In addition, the friction coefficients of the 45-steel substrate during the wear process were always larger than those of the flame-sprayed coatings and the laser-melted Ni60/WC coatings, and the average friction coefficients of the flame-sprayed and laser-melted coatings were 0.46 and 0.52, respectively, while the friction coefficients of the 45-steel substrate as 0.80 for the average values calculated for the 30 min wear process. The friction coefficient of the coating fluctuates with time, which is mainly due to the interaction of the hard-phase particles in the coating with the counter-abrasive steel balls [15]. It is noteworthy that laser-melted Ni60/WC coatings have significantly improved microhardness and a smaller coefficient of friction. Therefore, the preparation of Ni60/WC coatings on the surface of 45-steel plunger parts will significantly improve the wear resistance of the plungers during service.

The cross-sectional profiles of the laser cladding Ni60/WC coating, flame spray welding coating, and 45-steel substrate after wear are shown in Figure 8b. It can be found that the plunger surfaces of the two coatings have smaller volume wear under the same wear parameters. Measurements of the three-dimensional profiles after wear revealed that the wear profile widths of the laser cladding and flame-sprayed coatings were 628.2 and 645.2 μm, respectively, and the wear depths were 17.0 and 19.6 μm, respectively; however, the width and depth of the wear profile of the 45-steel substrate were 856.3 μm and 28.2 μm, respectively. The wear characteristics of the coatings and the 45-steel substrate were quantitatively analyzed from the wear profile measurements, and the coatings showed a significant increase in wear resistance compared to the substrate, with the laser cladding coatings having a slightly higher wear resistance than the flame-sprayed coatings. The volume loss of the coating was significantly lower compared to the 45-steel substrate, which is consistent with the trend of the coefficient of friction. The excellent wear resistance of the coatings can be attributed to the higher microhardness of the coatings, which further demonstrates the feasibility of adding hard phases to the coatings to improve the wear resistance of 45-steel plungers.

### 3.5. Corrosion Resistance

**Figure 9:**
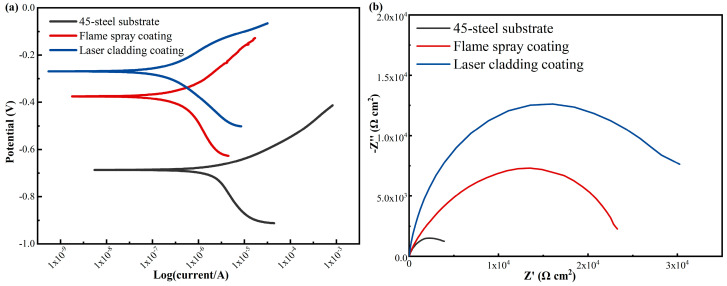
Polarization curves (**a**) and Nyquist plot (**b**) of Ni60/WC coating via laser cladding, flame-sprayed coatings, and 45-steel substrates.

**Figure 10:**
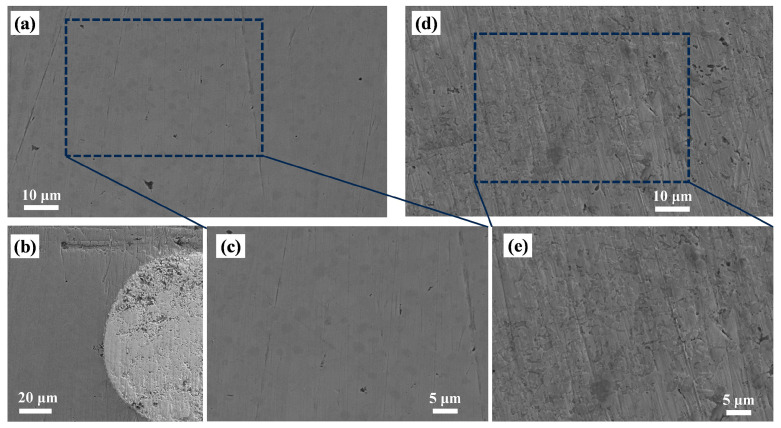
SEM micrographs of cross-sections after exposure to corrosion in the vicinity of (**a**,**c**) laser-melted coatings, (**b**) coated WC particles, (**d**,**e**) flame-sprayed coatings.

Polarization curves of the laser-melted Ni60/WC coating and 45-steel substrate in 3.5 wt.% NaCl solution are shown in Figure 9a, and the results obtained by fitting the polarization curves by Tafel extrapolation are summarized in Table 4. It can be found that the Ni60/WC coating has the smallest absolute value of corrosion potential Ecorr and has a lower self-corrosion current density Icorr than the substrate and flame-sprayed coating. The corrosion potentials of the laser cladding and flame-sprayed coatings were −0.270 and −0.375 V, respectively, and the corrosion current densities were 2.52 × 10^−7^ and 4.27 × 10^−7^ A/cm^2^, respectively. In contrast to the coatings, the corrosion potentials and corrosion current densities of the 45-steel substrate were −0.687 V and 3.99 × 10^−6^ A/cm^2^, respectively. The corrosion rates of the laser cladding coating, flame-sprayed coating, and 45-steel substrate were 2.96 × 10^−3^, 5.02 × 10^−3^, and 46.9 × 10^−3^ mm/year, respectively. Figure 10 shows SEM images of the coatings after exposure to corrosion in NaCl solution, and it can be found that the morphology of the laser cladding coatings after corrosion is smoother than that of the flame-sprayed coatings; both coatings exhibit the characteristics of uniform corrosion. The above results indicate that the laser-melted Ni60/WC coatings have higher corrosion resistance, which can inhibit the electrochemical corrosion phenomenon of the 45-steel substrate during the service period and improve the service life of the plungers.

**Figure 11:**
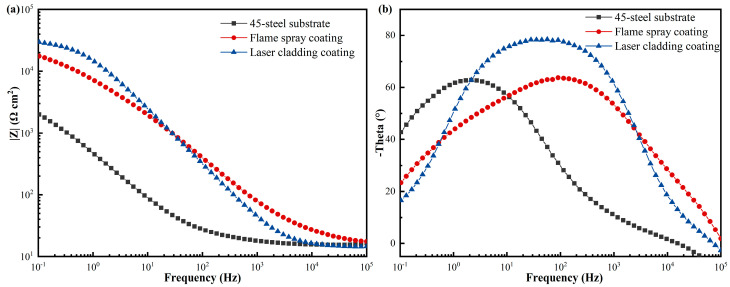
The magnitude–frequency (**a**) and phase–frequency plots (**b**) of laser cladding Ni60/WC coating, flame-sprayed coatings, and 45-steel substrates.

Nyquist and Bode plots of the laser cladding Ni60/WC coating, flame-sprayed coating, and 45-steel substrate are shown in Figure 9b and Figure 11, respectively, from which it can be observed that the coating has a significantly larger tolerant arc radius than the 45-steel substrate. The coating and the 45-steel substrate have incomplete tolerant arc radii, and the larger ones indicate higher corrosion resistance. The excellent corrosion resistance of the laser cladding Ni60/WC coating can be attributed to the superior corrosion resistance of the Ni-based alloy material over the Fe-based alloy material. According to the XRD results shown in Figure 5b, the coating is mainly composed of γ-(Ni, Fe) phase, and the 45-steel matrix is mainly composed of α-Fe phase. As a result, the Ni60/WC coatings prepared via the laser cladding technique on 45-steel have significantly improved corrosion resistance, with the coating surface providing a greater charge transfer impedance when corrosion behavior occurs. Figure 11 shows the Bode plots of the coatings and the 45-steel substrate, and it can be noticed that both materials exhibit a peak in the phase–frequency diagram, which further verifies that the laser-melted coatings are free of cracks and defects.

## 4. Conclusions

Ni60/WC coatings were prepared on the surface of 45-steel plungers via the laser cladding technique, and the microstructure and phase composition of the coatings were analyzed. Reciprocating friction and wear performance analysis was carried out according to the kinematic characteristics of the plunger in service, and 3.5 wt.% NaCl solution was selected as the corrosion solution to analyze the electrochemical behavior of the coating. The performance of the flame-sprayed plunger, which is widely used in the reciprocating pump, was compared to analyze the application potential of the laser cladding coating plunger. The main conclusions of this study are as follows:

1. The laser heat source irradiated the surface of the 45-steel plunger to form a liquid molten pool, which melted Ni60/WC powder and solidified rapidly, and the coating without defects, such as microcracks and porosity, was prepared in the plunger indication. The coatings prepared via laser cladding showed excellent metallurgical bonding with the substrate, and elemental analysis revealed that elemental diffusion occurred between the WC particles and the melted Ni60 alloy after irradiation by the laser heat source.
2. The Ni60/WC prepared via laser cladding is mainly composed of the γ-(Ni, Fe) phase, while the 45-steel matrix is mainly composed of the α-Fe phase, and the WC particles are only present in the interior of the coating, not on the surface. Microstructural analysis reveals that the top region of the coating is dominated by equiaxed crystals and equiaxed-like crystals, the middle region shows a typical dendritic crystal structure, and the bottom region of the cytosolic crystals shows the phenomenon of elongation along the perpendicular direction of the fusion line between the coating and the substrate; the grain size of the cytosolic crystals is significantly larger compared with that of the top region.
3. The highest microhardness value of the coating is 821.8 HV_0.5_, which is 310.9% higher compared to the 45-steel substrate (~200 HV_0.5_) and is superior to the microhardness of the flame-sprayed plunger coating of 545.5 HV_0.5_. The laser cladding coatings have a lower coefficient of friction than 45-steel substrates and flame-sprayed coatings and a lower volumetric wear rate over the same period of time. Both coatings showed uniform corrosion characteristics in the solution, and the surface of the laser cladding coating after corrosion was smoother than that of the flame-sprayed coating. The laser cladding coating had the highest wear resistance and excellent corrosion resistance. In addition, the laser cladding coating had a much smaller corrosion current density and corrosion rate of 3.5 wt.% NaCl solution, which is promising for reciprocating pump plunger applications.

## Figures and Tables

**Table 1:** Chemical composition of 45-steel substrate and Ni60 alloy powder (wt.%).

Elements	C	Si	Cr	Mn	Fe	B	Ni	Cu
45-steel substrate	0.45	0.2	0.24	0.5	Bal	-	0.22	0.25
Ni60 alloy powder	0.72	-	16.34	-	4.13	2.87	Bal	-

**Table 2:** Detailed process parameters for laser cladding of Ni60/WC coatings.

Parameters	Laser Power (W)	Spot Diameter (mm)	Axial Speed (mm/s)	Rotational Speed (r/min)	Powder Feeding Speed (g/min)	Argon Flow Rate (L/min)
Values	1700	3	0.14	5	17	25

**Table 3:** The chemical compositions corresponding to the different positions are labeled in Figure 5d.

Position	Elements (at.%)					
	C	W	Mn	Cr	Fe	Ni
A	52.35	47.65	-	-	-	-
B	58.39	41.61	-	-	-	-
C	26.93	1.16	0.37	11.56	48.76	10.78
D	22.49	0.73	2.41	4.10	47.70	22.55
E	27.03	1.11	0.36	8.15	47.10	16.24

**Table 4:** Electrochemical corrosion parameters of Ni60/WC coatings via laser cladding, flame sprayed coatings, and 45-steel substrates.

Samples	Corrosion Potential (Ecorr/V)	Corrosion Current Density (Icorr/Acm^−2^)	Corrosion Rate (mmPY)
45-steel substrate	−0.687	3.99 × 10^−6^	46.9 × 10^−3^
Flame spray coating	−0.375	4.27 × 10^−7^	5.02 × 10^−3^
Laser cladding coating	−0.270	2.52 × 10^−7^	2.96 × 10^−3^

## Data Availability

The datasets analyzed during the current study are available from the corresponding author on reasonable request.
